# *Pythiogeton manoomin*, a new species causing root and basal stalk rot of wild rice in the United States

**DOI:** 10.1080/21501203.2019.1635216

**Published:** 2019-06-30

**Authors:** Hung K. Doan, R. Michael Davis

**Affiliations:** Department of Plant Pathology, University of California, Davis, CA, USA

**Keywords:** Pythiaceae, Pythiogeton, root rot, wild rice, zizania, 1 new taxon

## Abstract

A new species of *Pythiogeton* isolated from wild rice exhibiting rot and dieback of roots and stalks in California is described. *Pythiogeton manoomin* sp. nov. is characterized by coenocytic hyphae, club-like appressorium, and terminal or intercalary sporangia, which are often a short distance from the end of supporting hyphae. The protoplasm is discharged through a discharge tube into an elongate transient vesicle, which soon disappears, leaving the naked protoplasm to differentiate into reniform zoospores. *Pythiogeton manoomin* also produces thick-walled pigmented chlamydospores, not found in other *Pythiogeton* species. In greenhouse trials, *Pg. manoomin* did not infect economically important crops such as rice, bean, chard, corn, carrot, lettuce, oat, radish, sweet pepper, tomato, or wheat. Phylogenetic analysis based on ITS data supports the conclusion that this organism is a new species that is most closely related to *Pg. ramosum*. In this paper, we describe morphological characteristics, temperature–growth relationships, pathogenicity, and phylogenetic relationships that support the description of this taxon as a new species, *Pythiogeton manoomin* sp. nov.

urn:lsid:zoobank.org:pub:4C63AAA4-4D4A-4679-A344-79B75121A5C6

## Introduction

The genus *Pythiogeton* (*Pg*.), together with *Pythium* and *Phytophthora*, belongs to the family *Pythiaceae* of the *Oomycota*. Minden first described members of the genus in 1916, and since then, *Pythiogeton* species have been reported in the USA (Drechsler 1932), Canada (Sparrow 1932), Germany and Denmark (Lund 1934), Japan (Ito and Tokunaga 1935), England (Sparrow 1936), China (Shen and Siang 1948), Africa (Gaertner 1954), India (Dayal 1968), Brazil (Beneke and Rogers 1970; Rocha et al. 2014), Taiwan (Watanabe 1974), Poland (Batko 1971), Pakistan (Lodhi et al. 2006), and Australia (Le et al. 2014). *Pythiogeton* species are characterized by production of zoospores outside of sporangia from a naked mass of protoplasm, not from a vesicle, which is characteristic of *Pythium* species. Members of the genus are facultative, anaerobic, ubiquitous inhabitants of soil and water. *Pythiogeton* can be readily isolated from plant materials submerged in stagnant water, and prefers anaerobic conditions (Huang et al. 2013).

**Table 1.** Averaged daily colony radial growth and standard deviation, in mm, on Rye B agar. Three plates were used for each temperature, and the experiment was repeated three times.

*Pythiogeton manoomin* isolates	Averages radial colony growth (mm day^−1^)								
	4°C	12°C	15°C	18°C	21°C	25°C	28°C	31°C	34°C
BV1	0.33 ± 0.58	4.00 ± 2.65	5.34 ± 3.87	12.11 ± 3.06	16.11 ± 1.93	23.44 ± 3.21	18.56 ± 3.03	7.22 ± 3.06	0.67 ± 0.58
BV2	0.67 ± 0.58	3.33 ± 2.65	5.11 ± 2.52	10.89 ± 3.21	15.44 ± 2.52	22.89 ± 2.79	18.11 ± 3.07	8.33 ± 2.00	0.67 ± 0.58
BV3	0.00 ± 0.00	2.89 ± 1.15	6.11 ± 2.52	11.89 ± 2.52	14.33 ± 2.00	22.56 ± 2.79	17.89 ± 2.51	7.78 ± 4.16	0.33 ± 0.58

To date, the *Pythiogeton* genus encompasses 15 recognized species: *Pythiogeton utriforme* (Minden 1916), *Pg. ramosum* (Minden 1916), *Pg. transversum* (Minden 1916), *Pg. autossytum* (Drechsler 1932), *Pg. uniforme* (Lund 1934), *Pg. dichotomum* (Ito and Tokunaga 1935), *Pg. nigrescens* (Batko 1971), *Pg. zeae* (Jee et al. 2000), *Pg. zizaniae* (Ann et al. 2006), *Pg. abundans* (Huang et al. 2012), *Pg. microzoosporum* (Huang et al. 2012), *Pg. oblongilobum* (Huang et al. 2012), *Pg. paucisporum* (Huang et al. 2012), *Pg. proliferatum* (Huang et al. 2012), and *Pg. puliensis* (Huang et al. 2012). Most described *Pythiogeton* species are saprophytic, with only three species, *Pg. zeae, Pg. zizaniae*, and *Pg. ramosum* known to be pathogenic to corn, water bamboo, and ginger, respectively (Jee et al. 2000; Ann et al. 2006; Le et al. 2014).

Wild Rice (*Zizania* spp.) or manoomin, as it is called in the Ojibwe language, is a semi-aquatic grass species endemic to North America and China. Three species of wild rice, *Zizania palustris, Z. aquatica*, and *Z. texana*, are native to North America, and one species, *Z. latifolia*, is native to China. Wild rice is one of America’s oldest indigenous cultivated grains, with over 90 percent of cultivated wild rice grown in the United States produced in California and Minnesota (Hayes et al. 1989; Oelke 1993; Steeves 1952). Wild rice-growing regions in California include Shasta, Lake, Modoc, Lassen, Butte, Colusa, Yuba, Yolo and Sutter counties. In the summer of 2012, an outbreak of a newly discovered root and basal stalk rot of wild rice (*Zizania palustris* L.) cv. Franklin was observed in a 16-ha field in Big Valley, Lassen County, California. Infected plants exhibiting rot and dieback of roots and stalks were in various stages of decline, including death. A pythiaceous fungus was consistently isolated from the diseased plant and was identified as *Pythiogeton* sp. (Doan et al. 2014). The purpose of this study is to characterize the new *Pythiogeton* species isolated from wild rice and to confirm its pathogenicity.

## Materials and methods

### Isolation and cultivation

Wild rice (*Zizania palustris* L.) cv. Franklin exhibiting rot and dieback of roots and stalks were collected from Big Valley, Lassen County, California (GPS coordinates 41°08′41.93″ N 121°10′07.49″ W). Symptomatic stem and root tissues from affected plants were placed on 9 cm Petri dishes (four pieces per plate) of PARP agar (17 g Difco corn meal agar containing 5 ppm pimaricin, 250 ppm ampicillin, 10 ppm rifampicin, and 100 ppm pentachloronitrobenzene), which were then incubated at 25°C in the dark for 1 week. Agar blocks 8 mm in diameter containing hyphal tips were transferred onto 9 cm Petri dishes (4 pieces per plate) of potato dextrose agar (PDA) and modified Rye B agar (RBA) containing 60 g rye-grain extract, 20 g of Difco Bacto Agar, and 20 g of sucrose as described by Caten and Jinks (1968).

### Colonial morphology and mycelial growth

The effect of temperature on mycelial growth was evaluated on Rye B agar for three *Pg. manoomin* isolates (BV1, BV2, and BV3) from wild rice. A 3 day-old, 8 mm-diameter Rye B agar plug of each isolate was transferred onto Rye B agar plates for each of the nine temperatures evaluated. The plates were incubated at 4, 12, 15, 18, 21, 25, 28, 31, or 34°C in the dark, and the growth rate was determined daily by measuring the linear colony diameter in three places up to 5 d for each temperature. Three plates were used for each treatment, and the experiment was repeated three times with at least 24 h between each experiment. The average growth rate for each isolate from the three replications was calculated for each temperature. The average growth rate of three isolates was tested using analysis of variance (ANOVA) in R version 3.3.2 (The R Foundation).

### Production of sporangia and release of zoospores

The morphology and development of sporangium were observed on Rye B agar using the method described by Chang (1988) and Huang et al. (2013). Hyphal tips were transferred onto modified Rye B agar. Agar blocks (5 x 5 x 3 mm) from 7 d-old Rye B agar cultures were placed in 9 cm Petri dishes (1 piece per plate) containing 20 mL of 10% clarified V-8 juice or deionized water and incubated in the dark at 24°C for 72 hours. The V-8 juice was then decanted and the mycelial mats were rinsed three times, at 20 min intervals, with sterile distilled water. For the development and release of zoospores, the mycelia mats with sporangia were incubated at 24°C for 48 h. Photographs were taken with a Leica DM5000 B microscope (Leica Microsystems, Wetzlar, Germany).

### Pathogenicity tests

Pathogenicity of *Pg. manoomin *to *Avena sativa* L. cv. Cat Grass (Lake Valley Seed, Boulder, CO), *Zea mays* L. cv. Golden Bantam Improved (Seed Savers Exchange, Decorah, IA), *Triticum aestivum* cv. Liquid Sunshine (Botanical Interests, Broomfield, CO), *Lactuca sativa* L. cv. Great Lakes Iceberg (Lake Valley Seed), *Beta vulgaris* subsp. *cicla* L. cv. Fordhook Giant (Burpee Garden Product Co., Warminster, PA), *Capsicum annum* L. cv. California Wonder (Burpee Garden Product Co.), *Daucus carota* subsp. *sativus* L. cv. Short ‘n Sweet (Burpee Garden Product Co.), *Solanum lycopersicum* L. cv. Moneymaker (Everwilde Farms, Sand Creek, WI), *Phaseolus vulgaris* L. cv. Ejote silvestre Contender (Burpee Garden Product Co.), *Raphanus sativus* L. cv. Cherry Belle (Burpee Garden Product Co.), *Oryza sativa* L. cv. Calrose (USDA # C.I. 8988), and *Zizania palustris* L. cv. Franklin was determined in the greenhouse. Ten seeds of each were planted in sterilize sand in plastic pots measuring 10 cm in diameter (750 ml). After 5 days, six 8 mm-diameter agar discs from the margin of a 7day-old culture growing on modified Rye B agar were placed in each pot. Pots inoculated with six 8 mm-diameter agar discs from a modified Rye B agar plate were used as controls. After 20 d post inoculation, root and crown tissues were examined for symptoms and disease was recorded. The experimental design was a randomized block with ten replications (blocks) in the greenhouse on a 13-h photoperiod provided by high pressure sodium bulbs with daytime temperatures ranging from 80–85°C and nighttime temperatures ranging from 65–70°C. The pathogenicity test was repeated three times. Re-isolation of the pathogen from crown and root tissues was attempted for all inoculated plants, as described above. Damping-off assay was performed as above with the exception that 10 seeds were planted into sterilized sand that was pre-inoculated with 12 8 mm-diameter agar discs from the margin of a 7day-old culture grown on modified Rye B agar. After 10 d post inoculation, germinated seeds were counted, and re-isolation of the pathogen from seeds was attempted, as described above.

### DNA extraction, PCR amplification, sequencing ITS rDNA

Total genomic DNA from three *Pythiogeton manoomin* isolates (BV1, BV2, and BV3) was extracted from mycelia using Qiagen® DNeasy Plant Mini Kit ™ (Valencia, CA) according to the manufacturer’s protocol. The internal transcribed spacer (ITS) region was amplified by PCR and sequenced using universal ITS5 and ITS4 primers (White et al. 1990). Three μL of genomic DNA were directly added to a 50 μL reaction that consisted of: 10 μL 5× Colorless GoTaq Reaction buffer (Promega Corp., Madison, Wisconsin), 5 μL 25 mM MgCl_2_, 4 μL containing 2.5 mM each dNTP, 0.2 μL GoTaq *Taq* polymerase (Promega Corp.), 0.1 μL each of a 50 μM concentration of primers, and 27.6 μL sterile deionized water. The thermal cycling parameters were initial denaturation at 94°C for 5 min followed by 34 cycles consisting of denaturation at 94°C for 1 min, annealing at 55°C for 2 min, and extension at 72°C for 2.5 min. A final extension at 72°C for 10 min was done at the end of the amplification followed by 4°C. The PCR products were purified with GeneJet PCR Purification Kit (Cat. No.: K0702, Thermo Fisher Scientific, Waltham, MA). Sequences of the forward and reverse strand were conducted by the College of Biological Sciences UCDNA Sequencing Facility (University of California, Davis, CA).

### Sequence alignment and phylogenetic analyses

DNA sequences were edited and assembled using the BioEdit software (http://www.mbio.ncsu.edu/BioEdit/bioedit.html). The ITS sequence for all three *Pythiogeton manoomin* isolates was identical. Therefore, the sequences of *Pythiogeton manoomin* isolate BV1 was submitted to GenBank (KF719169). The ITS sequence (JQ610201) of *Pythium aphanidermatum* was selected as an outgroup. Sequences were aligned using ClustalW, and a phylogenetic tree was constructed with MEGA 5.03 (Tamura et al. 2011), using the maximum parsimony method with 1000 bootstrap replication and evolutionary distance analyzed according to Tamura-Nei model (Tamura et al. 2011). The maximum parsimony tree was obtained using the Tree-Bisection-Regrafting algorithm with search level 5 in which the initial trees were obtained by the random addition of sequences (Nei and Kumar 2000). All positions containing gaps and missing data were eliminated. The aligned sequence data set was deposited in TreeBASE (No. 24,156).

## Taxonomy

**Figure 1.**
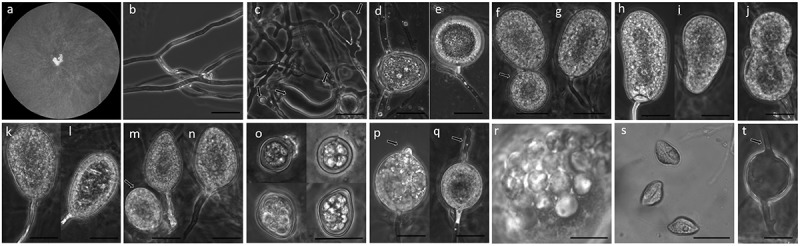
*Pythiogeton manoomin*. (a), Colony on PDA; (b–c), hyphae and appressoria (arrows) on RSA; (d–e), sporangia, terminal or intercalary and short distance from the end of supporting hyphae; (f–g), globose (arrow) and ellipsoid sporangia, (h–i), reniform sporangia, (j), bilobate sporangia, (k–l), pyriform sporangia, (m–n), globose (arrow) and ovoid sporangia; (o), chlamydospores, (p–q), sporangia initiating formation of the discharge tube (arrowheads), (r), formation of zoospores from a protoplasm mass, and, (s), discharged zoospores; (t), empty sporangia, showing empty discharge tubes (arrows). Scale bars = 20 μM.

***Pythiogeton manoomin*** H. K. Doan and R. M. Davis, sp. nov. Figure 1

MycoBank MB 830264

### 

#### Typification

United States, California: Lassen County. Isolate BV1, designated as the holotype, was recovered from root tissue of wild rice (*Zizania palustris* L.) cv. Franklin from a commercial agricultural field on 20 June 2012 and deposited in the University and Jepson Herbaria (UC 2060102), Berkeley, California. The living culture of the isolate was deposited and maintained at the Centraalbureau voor Schimmelcultures (CBS 145436), Fungal Biodiversity Centre, Utrecht, The Netherlands. Description of the species was submitted to MycoBank (MB 830264) and the ITS sequence was deposited in GenBank (GenBank Accession No. KF719169).

#### Etymology

manoomin, referring to wild rice in the Ojibwe language. Wild rice or manoomin, is an important native grain with spiritual and subsistence significance to many indigenous people of North America.

#### Description

Colonies on Rye B agar at 25°C with daily growth rate of 23.0 mm per day, with no distinct pattern on PDA and Rye B agar. Hyphae coenocytic, 5–7 mm in diameter. Appressoria knob-like, 3.3–5.9 × 7.7–10.2 μM, or club-like, 7.9–15.7 × 42.1–72.0 μM. Sporangia terminal or intercalary on supporting hyphae, abundant, various in shape, globose, 21–33 μM in diam., ellipsoid, 20–35 × 32–80 μM, reniform, 25–35 × 35–80 μM, bilobate, 30–50 × 50–80 μM, pyriform, 25–35 × 40–60 μM, or ovoid, 25–45 × 35–50 μM. Chlamydospores, various in size and shape, 10–15 μM in diameter, thick-walled, intercalary, pigmented (golden-brown). Discharge tube 5–100 μM long and 5 μM wide, protruding from different positions around sporangia. Encysted zoospores 15–25 μM in diameter. Sexual structures absent. Cardinal temperatures (Table 1) for growth: minimum, 12°C; optimum, 21°C; maximum, 31°C.

## Results

### Pathogenicity tests

**–** Pathogenicity of *Pg. manoomin* to wild rice was confirmed by inoculation followed by re-isolation. After 14 days, 84% of inoculated wild rice plants in all tests developed root and basal stalk rot, consistent with the symptoms observed in diseased wild rice in the field. No symptoms were observed on other inoculated plants. In addition, *Pg. manoomin* caused damping-off of 71% of wild rice seedlings tested. *Pythiogeton manoomin* was consistently re-isolated on PARP from symptomatic wild rice plants and seeds, but not from other inoculated plants or seeds and control plants, thus fulfilling Koch’s postulate. *Pythiogeton manoomin* did not infect the seedlings of Asian rice, bean, chard, corn, carrot, lettuce, oat, radish, sweet pepper, tomato, or wheat. All control plants grown in noninoculated soil remained healthy at the end of the experiment.

### Sequence analysis

**Figure 2.**
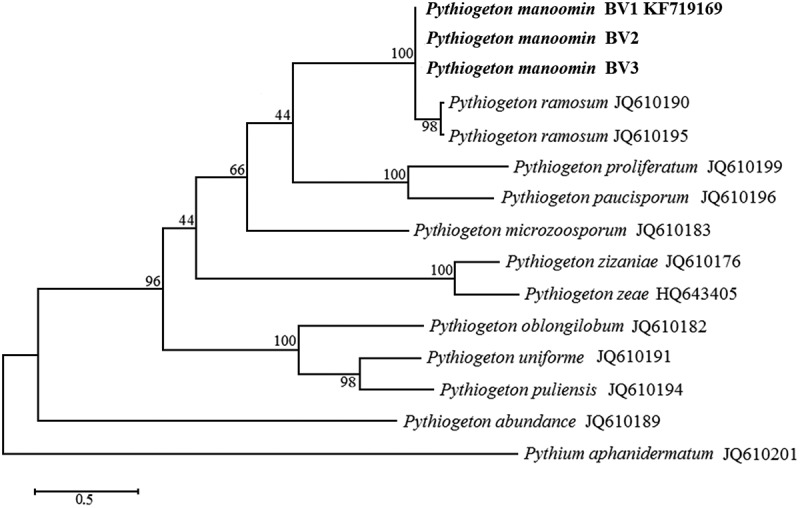
The Maximum Parsimony tree of Pythiogeton species with *Pythium aphanidermatum* as the outgroup based on ITS sequences. Numbers on the branches represent bootstrap values obtained from 1000 replications.

**–** The internal transcribed spacers (ITS) 1 and 2 flanking the 5.8S rRNA regions were amplified by PCR and sequenced using universal ITS5 and ITS4 primers. A BLAST search of the 855 bp sequences revealed 98% similarity with a sequence of *Pg. ramosum* isolate Pg-164 (GenBank Accession No. JQ610190.1). The 21 nucleotide differences suggest that the isolate from wild rice may be an unreported species. Based on previous published phylogeny, all *Pythiogeton* species belong to the same clade, separated from *Pythium, Phytophthora*, and downy mildew clades. Within the *Pythiogeton* clade, 10 subgroups (A1-A10) have been reported (Huang et al. 2013). The 21 nucleotide differences in the 5.8S rRNA region place *Pythiogeton manoomin* isolate BV1 into a new subgroup with high bootstrap value (Figure 2).

## Discussion

The genus *Pythiogeton* was erected in Germany by Minden in 1916. Since then, many species have been reported around the world (Jee et al. 2000). Of the described species, only three, *Pg. zeae, Pg. zizaniae*, and *Pg. ramosum*, are known to be plant pathogens (Jee et al. 2000; Ann et al. 2006; Le et al. 2014). Here, we characterized a new species of *Pythiogeton* capable of causing root and basal stalk rot of wild rice (*Zizania palustris* L.). *Pythiogeton manoomin* can be readily isolated from plant materials (in this case, wild rice) submerged in stagnant water during crop cultivation. Host range tests indicate that the pathogenicity of *Pg. manoomin* may be limited to wild rice and does not infect seedlings of economically important crops such rice, bean, carrot, chard, corn, lettuce, oat, radish, sweet pepper, tomato, or wheat. However, *Pg. manoomin* can possibly infect some wild grasses or weeds not tested here.

Morphologically, *Pythiogeton manoomin* is characterized by ovoid to ellipsoid sporangia in terminal or intercalary positions. Like all members of the genus *Pythiogeton, Pg. manoomin* sporangia release undifferentiating protoplasm through a discharge tube into an elongate transient vesicle, which soon disappears, leaving the naked protoplasm to differentiate into reniform zoospores. Appressoria form sparsely in contact with hard surfaces, (i.e. bottom surface of Petri dish). *Pythiogeton manoomin* is morphologically and genetically related to *Pg. ramosum*. Initially, descriptions of most *Pythiogeton* species were based exclusively on uncultured material (Minden 1916; Ito and Tokunaga 1935; Batko 1971; Jee et al. 2000). Isolation of some *Pythiogeton* species is difficult because other soilborne oomycetes outcompete it in culture. Recently, some species have been successfully grown in laboratory cultures (Jee et al. 2000; Ann et al. 2006; Huang et al. 2013). *Pythiogeton manoomin* and *Pg. ramosum* can be distinguished from other members of the genus by their readily culturable characteristics, as they can be cultured on a number of media, and have nonspecific requirements for growth media (Jee et al. 2000; Ann et al. 2006; Huang et al. 2013). In addition, both species are viable after subcultering and under storage conditions for up to two years (Jee et al. 2000; Le et al. 2015). The morphology of both species is variable and dependent on the type of water and medium used, so identification can be problematic if based on morphology alone (Le et al. 2015). However, *Pythiogeton manoomin* can be distinguished from *Pg. ramosum* based on pathogenicity and optimal growth temperature. *Pg. ramosum*, like other described *Pythiogeton* species, grows optimally at temperatures above 30°C, and does not grow at lower temperatures of 10–12°C (Huang et al. 2013; Le et al. 2015). In contrast, *Pg. manoomin* can grow at 10–12°C, with optimal growth at 25°C. Unlike other described *Pythiogeton* species, *Pg. manoomin* produces chlamydospores, which may allow for long-term survival (Jee et al. 2000). Chlamydospores are important survival structures of many plants of the family *Pythiaceae*, such as members of the *Pythium* and *Phytophthora* species (Hendrix and Campbell 1973; Mitchell 1978). *Pythiogeton manoomin* can also be distinguished from other *Pythiogeton* species based on pathogenicity tests. *Pg. ramosum* is pathogenic on ginger, bean, cauliflower, pepper, and lettuce (Le et al. 2015), *Pg. zeae* is pathogenic on corn and carrot (Jee et al. 2000), and *Pg. zizaniae* is pathogenic specifically to water bamboo, and does not infect seedlings of corn, rice, wheat, sorghum, cucumber, tomato, soybean or water spinach. (Ann et al. 2006). *Pythiogeton manoomin* causes disease only on wild rice among the crops tested here, but may be able to infect some wild grasses or weeds not tested here. Although *Py. manoomin* is phylogenetically similar to *Pg. ramosum*, a complete sequence comparison of the ITS revealed 98% similarity with a sequence of *Pg. ramosum* isolate Pg-164 (GenBank Accession No. JQ610190.1). However, the 21 nucleotide differences suggest that the isolate from wild rice is an unreported species.

Wild rice, the only cultivated cereal native to North America, is a staple food of indigenous peoples (Hayes et al. 1989; Oelke 1993). Today, wild rice is cultivated and commercialized for its unique flavor, texture, and nutritional value (Oelke et al. 1997). Several fungal diseases are considered important factors that limit wild rice production. For instance, fungal brown spots caused by *Bipolaris oryzae* (Bean and Schwartz 1961; Malvick and Percich 1993), sheath and stem rots caused by *Sclerotium hydrophilum* and *S. oryzae* (Punter et al., 1984), Fusarium head blight caused by *Fusarium graminearum* (Nyvall et al. 1999), crown and root rot caused by *Phytophthora erythroseptica* (Gunnell and Webster 1988), and damping-off caused by *Pythium torulosum* (Marcum and Davis 2006) have been associated with 100% losses in fields where disease was especially severe. This is the first report of the pathogenicity of a *Pythiogeton* species on wild rice. Infected plants exhibiting rot and dieback of roots and stalks were in various stages of decline, including death. Depending on disease severity, *Pg. manoomin* can cause losses in individual fields, and can vary from slight to near total stand failure. Since *Pg. manoomin* produces chlamydospores, management may be difficult. In conclusion, based on pathogenicity tests and evaluation of its morphological and molecular taxonomy, we confirm that *Pg. manoomin* (H. K. Doan and R. M. Davis), isolated from wild rice plants exhibiting rot and dieback of roots and stalks in California, is a distinct new *Pythiogeton* species.
